# Phosphodiesterase type 5 inhibitors related hearing impairment: a real world study based on the FDA adverse event reporting system

**DOI:** 10.1038/s41598-024-60493-w

**Published:** 2024-04-28

**Authors:** Xunyan Zhang, Lu Xia, Qiang Yang, Pingxiu Tang

**Affiliations:** 1 Department of Pharmacy, Suining Central Hospital, No.127, West Desheng Road, Chuanshan District, Suining, 629000 Sichuan People’s Republic of China; 2 Cancer Center of Suining Central Hospital, Suining, 629000 Sichuan People’s Republic of China

**Keywords:** Phosphodiesterase type 5 inhibitors (PDE5Is), FAERS, Hearing impairment, Disproportionality analysis, Adverse events, Health care, Risk factors, Urology

## Abstract

Recent studies focused on exploring phosphodiesterase type 5 inhibitors (PDE5Is)-related hearing impairment. This study aimed to comprehensively explore real-world hearing impairment associated with PDE5Is based on the US Food and Drug Administration Adverse Event Reporting System (FAERS). The characteristics and correlation of PDE5Is-related hearing impairment reported in the FAERS database from the fourth quarter of 2003 to the second quarter of 2023 were analyzed using disproportionality analysis. The Standardized Medical Dictionary for Regulatory Activities (MedDRA) Queries (SMQs) were used to analyze the adverse events (AEs) of hearing impairment. A total of 1,438 reported cases of hearing impairment were associated with four PDE5Is, revealing statistically significant reporting odds ratio (ROR), proportional reporting ratio (PRR), and information component (IC) with the SMQ. The average age of all patients was more than 55 years, over 70% of AEs were reported in men. Most of the reported cases were from the United States. Reports for all the drugs indicated an increase since 2008, except for avanafil. This study showed that the disability rates of PDE5Is were 8.14–40%, the rates of initial or prolonged hospitalization were 6.21–10.24%, and the rates of required intervention were 3.31–9.45%. The pharmacovigilance study identified a potential risk of hearing impairment associated with PDE5Is, indicating the need for continuous monitoring and appropriate management.

## Introduction

Phosphodiesterase type 5 inhibitors (PDE5Is) are a class of drugs widely used in the first-line treatment of erectile dysfunction (ED)^1^, which can stop the degradation and inactivation of cyclic guanosine monophosphate (cGMP) by selectively inhibiting the activity of PDE5. As a second messenger, the accumulation of cGMP in the penile corpora cavernosa during sexual stimulation induces the relaxation of penile smooth muscles and increases blood flow, finally resulting in penile erection^2^. Except for ED, many new applications of PDE5Is have been exploited, such as the treatment of male lower urinary tract symptoms (LUTS)^3^ and pulmonary arterial hypertension (PAH)^4^ for the abundance of PDE5 in all parts of the genitourinary tract and lung vascular smooth muscle.

As the first selective PDE5I, sildenafil was approved by the Food and Drug Administration (FDA) in 1998, Subsequently, other PDE5Is have been approved for marketing, including tadalafil, vardenafil, and avanafil^5^. Although PDE5Is share similar mechanisms of action, their pharmacokinetic properties differ. Avanafil is prominent for its shortest time to initiation of erection, the median maximum time is 30–45 min after dosing^6^. However, tadalafil shows the longest duration of action, and its half-life is about 17.5 h^7^. The variances in the onset and duration of action can cater to the needs and preferences of different patient.

The most commonly reported adverse events (AEs) of PDE5Is are flushing, headache, and dyspepsia^8^, which are mostly considered mild and self-limited without long-term deleterious consequences^9^. However, beyond the commonly reported AEs, some more infrequent but serious AEs associated with PDE5Is require a high level of attention, hearing impairment is one of the examples. Hearing loss is often considered an invisible disability, which not only significantly influences the long-term quality of life of patients, but also poses a public health burden on society^10^. The common reasons for acquired hearing loss include aging, exposure to noise or ototoxic drugs. Drug-related hearing impairment is often associated with antibiotics including aminoglycosides, glycopeptides and macrolides, platinum-based antineoplastics, loop diuretics, antimalarial drugs such as quinine, chloroquine, and non-steroidal anti-inflammatory drugs (NSAIDs)^11,12^. Since the initial report of sildenafil-related sudden sensorineural hearing loss in 2007^13^, additional statistics of PDE5Is-related hearing impairment from animal model^14^, and small or single-case series of patients^15^ have been published, raising further safety concern. However, due to the limited sample size of the trials, we need more real-world statistics from large-scale users to thoroughly discuss the characteristics of the drugs-related AE. To achieve this objective, we conducted a pharmacovigilance study based on the U.S. FDA Adverse Event Reporting System (FAERS) database to analyze the comprehensive reports of PDE5Is-related hearing impairment.

## Method

### Data source

The data for the research came from the U.S. Food and Drug Administration Public Data Open Project (Open FDA), whose raw data was imported from the FAERS database. OpenVigil 2.1, an open pharmacovigilance data extraction, mining, and analysis tool of the FAERS database was applied for the data extraction^16^, which operates only on cleaned FDA data, deleting most duplicates or reports with missing data.

AEs are coded using preferred terms (PTs) according to the Medical Dictionary for Regulatory Activities (MedDRA) (version 25.0) in FAERS. A specific PT can be assigned to several high-level terms (HLTs), high-level group terms (HLGTs), and system organ classes (SOCs). In addition, all PTs representing symptoms, signs, investigations, or diagnoses likely to be relevant can be grouped into meaningful categories using the Standardized MedDRA Queries (SMQs) to define a medical condition of interest. In this study, the narrow SMQ of PDE5Is-related hearing impairment included 56 PTs listing in Table 1.

**Table 1:** The PT term and code included in the narrow SMQ of hearing impairment.

The term of PT	Code	The term of PT	Code
Acoustic stimulation tests abnormal	10000526	Middle ear adhesions	10027582
Altered pitch perception	10075083	Middle ear effusion	10062545
Audiogram abnormal	10003761	Middle ear inflammation	10065838
Auditory disorder	10003778	Misophonia	10079388
Auditory recruitment	10003789	Mixed deafness	10027757
Autophony	10048827	Myringitis	10061302
Barotitis media	10004129	Neonatal deafness	10080897
Bone anchored hearing aid implantation	10070723	Neonatal hypoacusis	10080902
Cochlea implant	10009830	Neurosensory hypoacusis	10067587
Conductive deafness	10010280	Noninfective myringitis	10078830
Deafness bilateral	10052556	Ossicle disorder	10061327
Deafness neurosensory	10011891	Otoacoustic emissions test abnormal	10063643
Deafness occupational	10011893	Otosalpingitis	10033102
Deafness permanent	10011894	Otosclerosis	10033103
Deafness transitory	10011900	Ototoxicity	10033109
Deafness unilateral	10048812	Paracusis	10085733
Deafness	10011878	Presbyacusis	10036626
Diplacusis	10013032	Rinne tuning fork test abnormal	10039191
Dysacusis	10049712	Sudden hearing loss	10061373
Electrocochleogram abnormal	10014399	Tinnitus retraining therapy	10084652
Eustachian tube disorder	10061462	Tinnitus	10043882
Eustachian tube dysfunction	10015543	Tympanic membrane atrophic	10045208
Eustachian tube obstruction	10015544	Tympanic membrane disorder	10062218
Haematotympanum	10063013	Tympanic membrane perforation	10045210
Hearing aid therapy	10075385	Tympanic membrane scarring	10063604
Hearing therapy	10087034	Tympanometry abnormal	10045215
Hyperacusis	10020559	Tympanosclerosis	10045218
Hypoacusis	10048865	Weber tuning fork test abnormal	10047878

### Data extract

The retrospective pharmacovigilance study covered data in the FAERS database from the fourth quarter of 2003 to the second quarter of 2023. FAERS was queried by using the generic names “sildenafil” “vardenafil” “tadalafil” and “avanafil” with primary suspect, after removing duplicate reports (with the same ISR number), the reports were analyzed. Two researchers used SMQ-narrow and PTs to classify PDE5Is-related hearing impairment and extracted patient and drug information from reports.

### Signal detection

According to the basic principles of Bayesian and nonproportional analysis, reporting odds ratio (ROR)^17^, proportional reporting ratio (PRR)^18^, and Bayesian confidence propagation neural network (BCPNN) algorithms^19^ were applied to explore the association between PDE5Is and the AE of hearing impairment. The equations and criteria for the three algorithms are shown in Table 2, any of the three algorithms met the criteria, it would be considered as a positive signal.

**Table 2:** Summary of major algorithms applied for signal detection.

Algorithms	Equation^#^	Criteria
ROR	ROR = (a/b)/(c/d) 95%CI =e^ln (ROR)±1.96(1/a+1/b+1/c+1/d)^0.5^	95% CI > 1, N ≥ 2
PRR	PRR = (a/(a + c))/(b/(b + d)) χ^2^ = ∑[(O-E)2/E], [O = a, E = ( a + b )( a + c) /( a + b + c + d )]	PRR ≥ 2, χ^2^ ≥ 4, N ≥ 3
BCPNN	IC = log_2_^a(a+b+c+d)/((a+c)(a+b))^ IC025 =e^ln (IC)−1.96(1/a+1/b+1/c+1/d)^0.5^	IC025 > 0

^#^a: the count of reports containing both the suspect drug and the suspect adverse events. b: the count of reports containing the suspect adverse events with other medications (except for the drug of interest). c: the count of reports containing the suspect drug with other adverse events (except for the event of interest). d: the count of reports containing other medications and other adverse events.

ROR, reporting odds ratio; CI, confidence interval; N, the number of co-occurrences; PRR, proportional reporting ratio; χ^2^, chi-squared; BCPNN, Bayesian confidence propagation neural network; IC, information component; IC025, the lower limit of the 95% two-sided CI of the IC.

### Statistical analysis

We conducted a descriptive analysis to aggregate the clinical characteristics of patients with PDE5Is-related hearing impairment. The continuous variable was represented by mean ± standard deviation (SD), and the count variable was displayed as percentage. Pearson’s chi-square test or Fisher’s exact test with Bonferroni corrections was utilized to compare statistic of rates between different PDE5Is. All statistical analyses were conducted by IBM^®^ SPSS^®^ Statistics (version 26). *P* value < 0.05 were considered statistically significant.

## Results

### Descriptive analysis

A total of 86,848 AEs of drug-related hearing impairment were reported. The reported cases of 4 PDE5Is were 1438 during the fourth quarter of 2003 to the second quarter of 2023. The clinical characteristics of patients with PDE5Is-related hearing impairment are depicted in Table 3. The average age of all patients was more than 55 years, the highest proportion was for patients aged 45–74 years. Over 70% of AEs were reported in men, aligning with the intended use of PDE5Is, primarily for treating ED. The most reported cases were from the United States. Most cases related to sildenafil use were reported during 2008–2017, those related to vardenafil use during 2008–2012, and those related to tadalafil and avanafil during 2013–2023.

**Table 3:** The clinical characteristics of patients with PDE5Is-related hearing impairment.

Characteristics	Reports (N, %)			
	Sildenafil	Vardenafil	Tadalafil	Avanafil
N	786	127	515	10
Age				
≤ 44 years	54 (6.87%)	15 (11.81%)	36 (6.99%)	–
45–59 years	89 (11.32%)	36 (28.35%)	95 (18.45%)	4 (40.00%)
60–74 years	175 (22.26%)	25 (19.69%)	110 (21.36%)	4 (40.00%)
≥ 75 years	88 (11.20%)	5 (3.94%)	45 (8.74%)	–
Unknown	380 (48.35%)	46 (36.22%)	228 (44.27%)	2 (20.00%)
Mean ± SD	61.45 ± 12.61	55.96 ± 10.66	59.97 ± 11.15	58.13 ± 6.63
Gender				
Male	558 (70.99%)	125 (98.43%)	390 (75.73%)	10 (100.00%)
Female	150 (19.08%)	–	118 (22.91%)	–
Unknown	78 (9.92%)	2 (1.57%)	7 (1.36%)	–
Reported countries				
United States	651 (82.82%)	104 (81.89%)	421 (81.75%)	6 (60.00%)
Canada	29 (3.69%)	3 (2.36%)	12 (2.33%)	–
Great Britain	26 (3.31%)	1 (0.79%)	7 (1.36%)	–
Brazil	4 (0.51%)	4 (3.15%)	5 (0.97%)	–
Japan	11 (1.40%)	/	18 (3.50%)	–
Reported years				
2003–2007	46 (5.85%)	29 (22.83%)	30 (5.83%)	–
2008–2012	290 (36.90%)	93 (73.23%)	88 (17.09%)	–
2013–2017	254 (32.32%)	5 (3.93%)	256 (49.71%)	4 (40.00%)
2018–2023	196 (24.94%)	–	141 (27.38%)	6 (60.00%)

Besides, the information on the dosage, indication/comorbidity and concomitant/prior medicines of reported patients are summarized in Table 4. Consistent with the approved indications, the PDE5Is were mainly used for ED, PAH and benign prostatic hyperplasia. The dosage of the four drugs was also within the recommended dose range. Other comorbidities included hypertension, cardiac disorder, gastroesophageal reflux disease, and so on. Anticoagulants, diuretics, proton pump inhibitors, NSAIDs, and antihypertensive and lipid-lowering drugs were the most often reported concomitant/prior-used medicines, consistent with the reported comorbidities.

**Table 4:** The dosage, diseases and medicines information of patients with PDE5Is-related hearing impairment.

Characteristics	Reports (N)			
	Sildenafil	Vardenafil	Tadalafil	Avanafil
Dosage	100 mg, once (94)	10 mg, once (37)	20 mg, once (119)	100 mg, q4-6w (2)
	20 mg, tid (78)	20 mg, once (23)	5 mg, once (90)	100 mg, once (2)
	50 mg, once (62)	5 mg, once (4)	40 mg, once (46)	200 mg, once (1)
Indication/comorbidity	Erectile dysfunction (209)	Erectile dysfunction (76)	Erectile dysfunction (202)	Erectile dysfunction (7)
	Pulmonary arterial hypertension (118)	Gastrooesophageal reflux disease (2)	Pulmonary arterial hypertension (109)	Angina unstable (1)
	Hypertension (19)	Hypertension (2)	Benign prostatic hyperplasia (43)	Blood cholesterol increased (1)
	Cardiac disorder (17)	Prostatic disorder (2)	Hypertension (9)	Ischaemia (1)
	Gastrooesophageal reflux disease (14)	Sexual dysfunction (1)	Prostatic disorder (7)	–
Concomitant/prior medicines	Warfarin (35)	Clonazepam (11)	Ambrisentan (66)	Amlodipine (2)
	Furosemide (34)	Pantoprazole (11)	Selexipag (53)	Olmesartan (2)
	Spironolactone (26)	Fenofibrate (10)	Treprostinil (46)	Bisoprolol (1)
	Omeprazole (23)	Acetaminophen/hydrocodone (9)	Sildenafil (26)	Clopidogrelclopidogrel (1)
	Ambrisentan (21)	Duloxetine (9)	Furosemide (18)	Eszopiclone (1)
	Aspirin (20)	Protriptyline (9)	Warfarin (18)	Rosuvastatin calcium (1)
	Metoprolol (20)	Topiramate (9)	Amlodipine (13)	–
	Levothyroxine (18)	Tadalafil (5)	Acetylsalicylic acid (11)	–
	Acetylsalicylic acid (17)	Acetylsalicylic acid (4)	Aspirin (11)	–
	Bosentan (17)	Simvastatin (4)	Tamsulosin (11)	–

### Disproportionality analysis

The number of reports on hearing impairment related to the use of sildenafil, vardenafil, tadalafil, and avanafil was 786, 127, 515, and 10, respectively. The narrow SMQ of hearing impairment related to the four aforementioned PDE5Is were analyzed individually. According to the standards of the three algorithms, all of these four drugs exhibited statistically significant ROR, PRR, and information component (IC) values (Table 5). Among these, avanafil demonstrated the highest association with hearing impairment based on the highest signal strength [PRR = 6.34, χ^2^ = 40.08; ROR = 6.58, 95% two-sided confidence interval (CI) = 3.49–12.39; IC = 2.31, IC025 = 1.23]. Tadalafil followed avanafil (PRR = 3.43, χ^2^ = 886.77; ROR = 3.49, 95% two-sided CI = 3.2–3.81; IC = 1.73, IC025 = 1.59). And then vardenafil (PRR = 3.17, χ^2^ = 187.88; ROR = 3.22, 95% two-sided CI = 2.7–3.84; IC = 1.61, IC025 = 1.32), Sildenafil had the lowest association with hearing impairment (PRR = 2.89, χ^2^ = 969.16; ROR = 2.93, 95% two-sided CI = 2.73–3.15; IC = 1.48, IC025 = 1.37).

**Table 5:** Signal strength of PDE5Is-related hearing impairment.

Drugs	N	PRR (χ^2^)	ROR (95% two-sided CI)	IC (IC025)
Sildenafil	786	2.89 (969.16)	2.93 (2.73–3.15)	1.48 (1.37)
Vardenafil	127	3.17 (187.88)	3.22 (2.7–3.84)	1.61 (1.32)
Tadalafil	515	3.43 (886.77)	3.49 (3.2–3.81)	1.73 (1.59)
Avanafil	10	6.34 (40.08)	6.58 (3.49–12.39)	2.31 (1.23)

**Figure 1:**
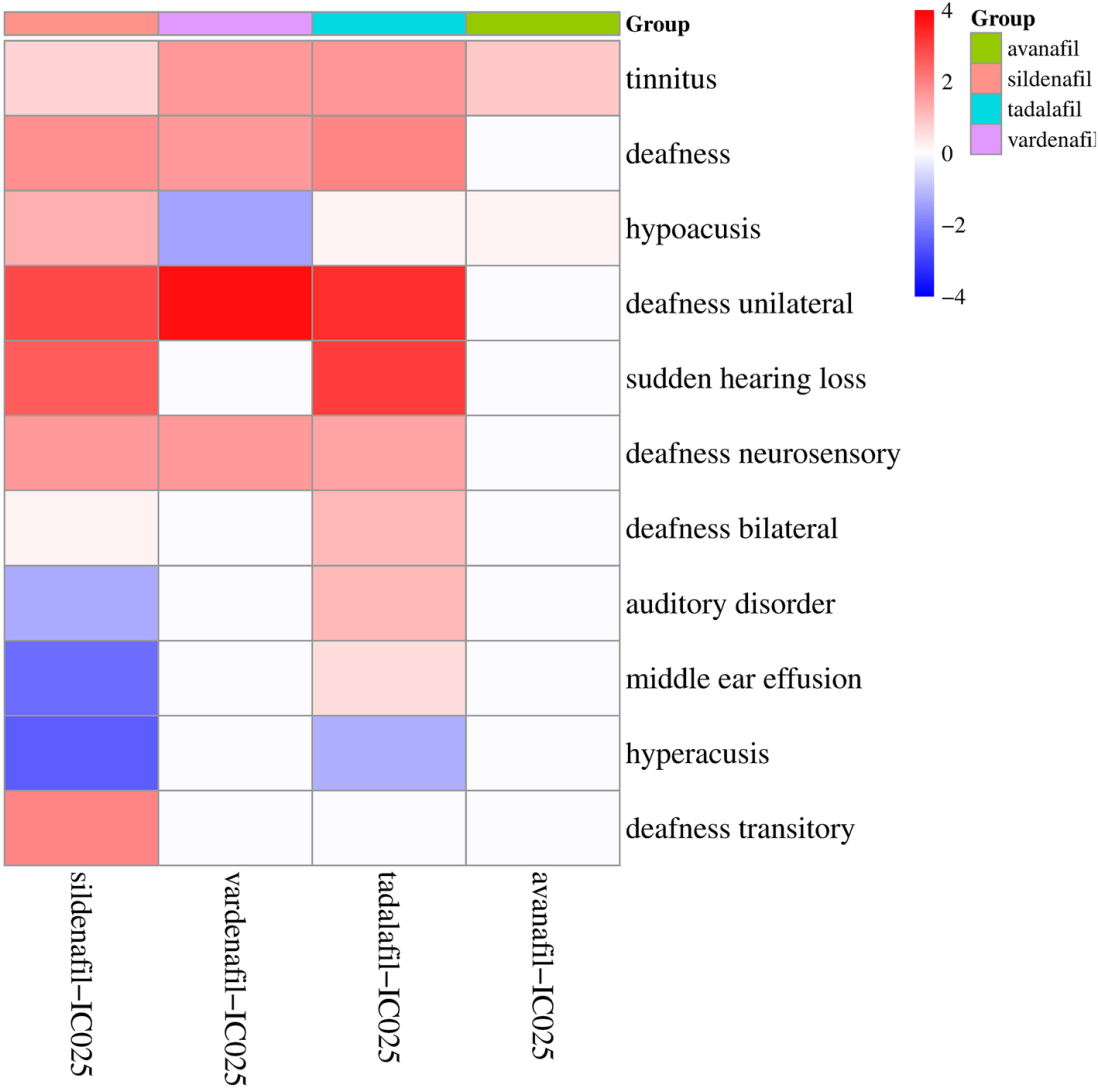
The correlation between PDE5Is and PTs included in the narrow SMQ of hearing impairment.

The signal strength of PTs under the narrow SMQ of hearing impairment was also analyzed (Table 6 and Fig. 1). Hypoacusis was the most reported PT for sildenafil (309 reports), whereas tinnitus was the most reported PT for vardenafil (53 reports), tadalafil (183 reports) and avanafil (5 reports). In terms of signal strength, sudden hearing loss showed the highest correlation with sildenafil (PRR = 10.55, ROR = 10.56) and tadalafil (PRR = 16.06, ROR = 16.08), vardenafil was associated with a higher risk of deafness unilateral (PRR = 27.15, ROR = 27.33), and avanafil was related to the risk of tinnitus (PRR = 10.44, ROR = 10.64).

**Table 6:** The signal strength of PTs under the narrow SMQ of hearing impairment (N ≥ 3).

Adverse event	Sildenafil			Vardenafil			Tadalafil			Avanafil		
	N	PRR (χ^2^)	ROR (95%CI)	N	PRR (χ^2^)	ROR (95%CI)	N	PRR (χ^2^)	ROR (95%CI)	N	PRR (χ^2^)	ROR (95%CI)
Tinnitus	169	2.04 (88.3)	2.05 (1.76–2.38)	53	4.36 (133.9)	4.39 (3.35–5.75)	183	4.02 (409.99)	4.04 (3.49–4.68)	5	10.44 (33.81)	10.64 (4.39–25.8)
Deafness	224	4.12 (520.9)	4.14 (3.63–4.73)	39	4.86 (115.65)	4.89 (3.57–6.7)	146	4.86 (441.11)	4.89 (4.15–5.76)	3	9.48 (15.09)	9.59 (3.07–29.96)
Hypoacusis	309	2.88 (374.45)	2.89 (2.59–3.24)	12	0.76 (0.7)	0.76 (0.43–1.34)	90	1.51 (15.2)	1.52 (1.23–1.87)	4	6.42 (13.32)	6.51 (2.43–17.5)
Deafness unilateral	104	10.26 (833.35)	10.29 (8.46–12.51)	41	27.15 (994.36)	27.33 (20.06–37.22)	80	14.24 (948.49)	14.29 (11.44–17.84)	–	–	–
Sudden hearing loss	38	10.55 (308.7)	10.56 (7.64–14.59)	–	–	–	32	16.06 (425.18)	16.08 (11.32–22.86)	–	–	–
Deafness neurosensory	30	5.07 (92.6)	5.08 (3.54–7.28)	9	10.3 (66.24)	10.31 (5.35–19.86)	18	5.5 (61.23)	5.5 (3.46–8.75)	–	–	–
Deafness bilateral	9	2.89 (9.19)	2.89 (1.5–5.56)	–	–	–	10	5.83 (35)	5.84 (3.13–10.88)	–	–	–
Auditory disorder	4	1.53 (0.3)	1.53 (0.57–4.09)	–	–	–	9	6.3 (34.64)	6.31 (3.27–12.16)	–	–	–
Middle ear effusion	3	0.85 (0)	0.85 (0.27–2.64)	–	–	–	8	4.14 (15.94)	4.14 (2.07–8.31)	–	–	–
Hyperacusis	4	0.6 (0.69)	0.6 (0.23–1.6)	–	–	–	5	1.37 (0.19)	1.37 (0.57–3.29)	–	–	–
Deafness transitory	17	8.67 (104.93)	8.67 (5.36–14.05)	–	–	–	–	–	–	–	–	–

### Outcome of PDE5Is-related hearing impairment

**Figure 2:**
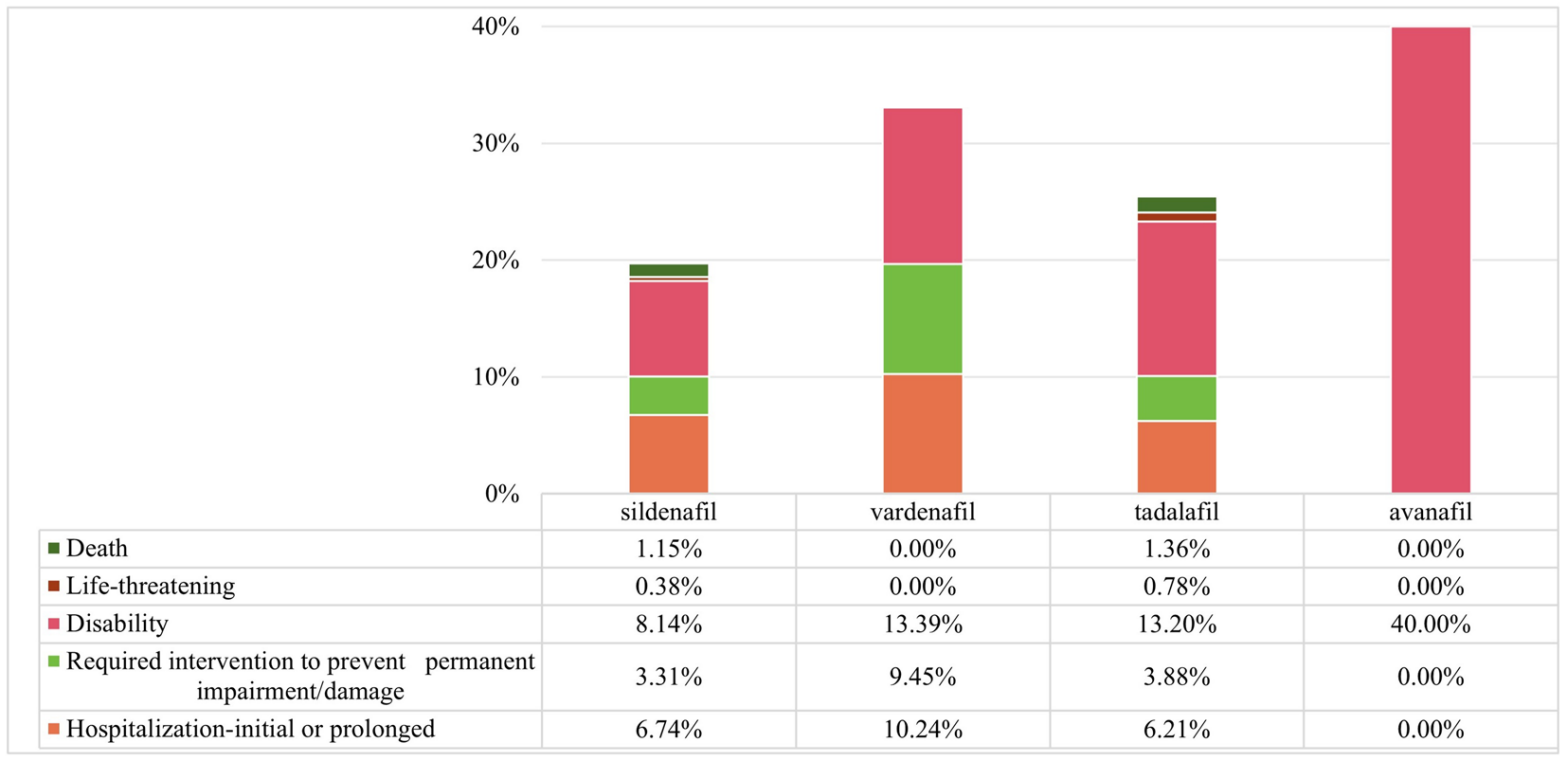
The reported outcome of PDE5Is-related hearing impairment.

We performed a statistical analysis of the outcomes recorded in the database to analyze the prognosis of PDE5Is-related hearing impairment. The proportions of hospitalization-initial or prolonged, and required intervention to prevent permanent impairment/damage, disability, life-threatening, and death were calculated (Fig. 2). The rates of hospitalization, life-threatening, and death, showed no significant differences (Pearson’s chi-squared test, *P* > 0.05) among PDE5Is containing the aforementioned outcomes. Avanafil exhibited the highest rate of disability (40%) among the five PDE5Is, whereas sildenafil had the lowest rate (8.14%), significant differences were found between sildenafil and avanafil/tadalafil (Bonferroni-corrected test, *P* < 0.05). The rate of required intervention to prevent permanent impairment/damage caused by vardenafil was 9.45%, which was significantly higher than for sildenafil (3.31%) and tadalafil (3.88%) (Bonferroni-corrected test, *P* < 0.05).

## Discussion

In this study, cases of PDE5Is-related hearing impairment were more prevalent among middle-aged and elderly men (average age > 55 years). Apart from avanafil (introduced in 2012), all drugs showed an increase in the number of cases since 2008. An important factor contributing to this trend could be the assessment conducted by the FDA Drug Safety Newsletter regarding the reports of PDE5Is-related hearing impairment. In 2008, the FDA urged for the revision of labeling for this class of drugs to include the potential risk of developing sudden hearing loss. The reported patients primarily used concomitant or prior medications such as anticoagulants, diuretics, proton pump inhibitors, NSAIDs, antihypertensive and lipid-lowering drugs. The loop diuretics and NSAIDs were often associated with the risk of hearing impairment, their influence on the AEs could not be ruled out. However, selecting cases only labeled "primary suspect" of PDE5Is would make the results more reliable. Besides, the ratio of using loop diuretics or NSAIDs as co-medication was relatively low: 9.03% for sildenafil, 10.24% for vardenafil, and 7.77% for tadalafil. In terms of dosage, for ED^20^, the recommended dose is 25–100 mg for sildenafil, 5–20 mg for vardenafil and tadalafil, and 50–200 mg for avanafil. While for PAH^21^, the recommended dose was 20 mg tid for sildenafil, 5–10 mg bid for vardenafil, and 20–40 mg qd for tadalafil. The reported dose of the four drugs was mostly within the recommended dose range, however, a prospective observational study conducted in 2013^15^ showed that three patients taking 20 mg of tadalafil experienced a significant increase in their hearing thresholds in the higher frequencies, compared with those taking 10 mg of tadalafil. Whether higher dosage is associated with the AEs of hearing loss remains unknown. Most of indications were consistent with the approved indications, however, confirming whether the off-label use of the drugs proved to be difficult for comorbidity was included.

The data mining results revealed that four PDE5Is were associated with the SMQ of hearing impairment. When examining the correlation of each drug with PTs under the SMQ, PDE5Is exhibited a significant association with deafness unilateral, sudden hearing loss and tinnitus. The most commonly reported PTs were hypoacusis and tinnitus, other highly reported PTs involved deafness, deafness unilateral, which was consistent with the published literature. Manna et al. summarized the clinical symptoms of case reports involving PDE5Is-related hearing impairment, they found that 75% of the cases indicated unilateral deficits, while 22% reported tinnitus^22^. Drugs-related hearing impairment usually indicates poor prognosis, which is the same for PDE5Is. A review of FDA post-marketing data in 2009 revealed that only 32% of patients who reported PDE5Is-related hearing impairment had documented improvement in their hearing from the initial presentation^23^. The study showed that the disability rates related PDE5Is were 8.14–13.39%, although that of avanafil was as high as 40%, the low number of reports may reduce credibility. The rates of hospitalization or prolonged were 6.21–10.24%, and the rates of required intervention were 3.31–9.45%. These findings underscored the necessity to pay more attention to PDE5Is-related hearing impairment. A large number of factors might lead to a poor outcome, and hence establishing causal relationships between the use of a certain drug and poor prognosis is difficult. The number of cases for avanafil was 10, which was relatively small compared with the other three drugs. The characteristics and association of avanafil-related hearing loss might not be as reliable as those for other PDE5I. For instance, the associations between PDE5Is and patient prognosis need to be verified through more high-quality prospective studies.

Despite these scattered reports and studies, the exact mechanisms behind PDE5Is-related hearing impairment remain unclear. The predominating theories focus on the nitric oxide (NO)/cGMP pathway. NO has been demonstrated to exist in the cochlear vascular endothelium and is linked to cochlear damage^24^, PDE5Is can increase the release of NO by inhibiting the degradation of cGMP. Besides, the accumulation of intracellular cGMP activates nuclear factors-kappa beta (NF-kB)^25^ and mitogen-activated protein (MAP) kinase. NF-kB has been postulated as a possible causative agent of sudden sensorineural hearing loss^26^. The inhibition of c-Jun-K-terminal kinases (JNK) and p38^27^, two important isoforms of MAP, is associated with the alleviation of ototoxic stress and the promotion of otoprotective effects of cochlear hair cell^28^. No specific treatment is available for PDE5Is-related hearing impairment, systemic steroids serve as the most common choice^29^.

Although this study has some advantages in terms of real-world research and data mining techniques, there are some limitations. First, as a spontaneous reporting system, the reports in the FAERS database might be inevitably under-reported, incomplete reported, and false or inaccurate reported. Second, we could acquire statistics on the basic information of many patients, however, the underlying disease status and accurate medication history for patients were not clear, leading to confounding factors and uncertainty in our analysis. Third, disproportionality analysis was highly sensitive to statistical association analysis, but we couldn’t confirm the causal relationship between the specific AEs and drugs. Fourth, the correlation between the prognosis and the use of PDE5Is was not certain, especially for death, which might be more related to the underlying basic disease. Despite the aforementioned drawbacks, our study provided valuable information on the safety risks associated with hearing impairment among PDE5Is, Clinicians should remain vigilant about the potential risk of hearing impairment when prescribing these drugs in clinical practice. Besides, this study might provide a novel basis for further well-organized clinical investigations into hearing impairment related to PDE5Is.

## Conclusions

The analysis of reports from FAERS database showed that PDE5Is were possibly associated with the AEs of hearing impairment. The study also provided a comprehensive overview of the clinical characteristics and prognosis of PDE5Is-related hearing impairment, warranting continued surveillance and proper management for patients using PDE5Is. Further prospective studies involving large-scale users are needed to confirm this association.

## Data Availability

The data and materials used in this study can be available from the corresponding author on reasonable request.

## Code availability

Available on reasonable request from the corresponding author.
